# Transgenic Mice Overexpressing the Divalent Metal Transporter 1 Exhibit Iron Accumulation and Enhanced Parkin Expression in the Brain

**DOI:** 10.1007/s12017-017-8451-0

**Published:** 2017-07-10

**Authors:** Cheng-Wu Zhang, Yee Kit Tai, Bing-Han Chai, Katherine C. M. Chew, Eng-Tat Ang, Fai Tsang, Bryce W. Q. Tan, Eugenia T. E. Hong, Abu Bakar Ali Asad, Kai-Hsiang Chuang, Kah-Leong Lim, Tuck Wah Soong

**Affiliations:** 1 National Neuroscience Institute, 11 Jalan Tan Tock Seng, Singapore, 308433 Singapore; 2 Department of Physiology, Yong Loo Lin School of Medicine, National University of Singapore, Block MD9, 2 Medical Drive, Singapore, 117597 Singapore; 3 Singapore Bioimaging Consortium (SBIC), A*STAR, Singapore, 138667 Singapore; 4 Duke-NUS Medical School, Singapore, 169857 Singapore; 5 NUS Graduate School for Integrative Science and Engineering, Singapore, 117456 Singapore; 6 LSI Neurobiology/Ageing Programme, NUS, Singapore, 117456 Singapore; 7 Institute of Advanced Materials, Nanjing Tech University, Nanjing, 211816 People’s Republic of China

**Keywords:** 6-OHDA, Dopamine, Oxidative stress, Parkin, Parkinson’s disease

## Abstract

Exposure to divalent metals such as iron and manganese is thought to increase the risk for Parkinson’s disease (PD). Under normal circumstances, cellular iron and manganese uptake is regulated by the divalent metal transporter 1 (DMT1). Accordingly, alterations in DMT1 levels may underlie the abnormal accumulation of metal ions and thereby disease pathogenesis. Here, we have generated transgenic mice overexpressing DMT1 under the direction of a mouse prion promoter and demonstrated its robust expression in several regions of the brain. When fed with iron-supplemented diet, DMT1-expressing mice exhibit rather selective accumulation of iron in the substantia nigra, which is the principal region affected in human PD cases, but otherwise appear normal. Alongside this, the expression of Parkin is also enhanced, likely as a neuroprotective response, which may explain the lack of phenotype in these mice. When DMT1 is overexpressed against a Parkin null background, the double-mutant mice similarly resisted a disease phenotype even when fed with iron- or manganese-supplemented diet. However, these mice exhibit greater vulnerability toward 6-hydroxydopamine-induced neurotoxicity. Taken together, our results suggest that iron accumulation alone is not sufficient to cause neurodegeneration and that multiple hits are required to promote PD.

**Electronic supplementary material:**

The online version of this article (doi:10.1007/s12017-017-8451-0) contains supplementary material, which is available to authorized users.

## Introduction

Parkinson disease (PD) is a prevalent neurodegenerative movement disorder that is characterized pathologically by the rather selective loss of dopaminergic (DA) neurons in the substantia nigra pars compacta (SNpc). Although most cases of PD occur in a sporadic manner, a subset of PD cases is inheritable and attributable to mutations in specific genes (Chai and Lim 2013). For example, mutations in *α*-*synuclein* and *Parkin* are causative of autosomal dominant and recessive forms of PD, respectively. Notwithstanding this, it is important to recognize that even in individuals harboring PD-linked mutations, it typically takes several decades for the disease to surface, suggesting that additional factors (likely present in the environment) are involved in the pathogenesis of PD. Notably, exposure to metals such as iron and manganese has consistently been implicated by epidemiological studies to increase the risk for PD, particularly in view of their preferential accumulation in the SN and their role in promoting harmful oxidative reactions (Dexter et al. 1989). Moreover, aging, which is an unequivocal risk factor for PD, promotes iron accumulation in the brain. Notably, Zecca and colleagues reported that iron concentration increases linearly with age in the SN, while the amount in the locus coeruleus remains comparatively much lower even in individuals aged 80 and above (Zecca et al. 2004). However, because iron and manganese are both essential to human physiology, their levels are normally regulated by homeostatic mechanisms that constantly adjust the intake and excretion rates to avoid intoxication (or deficiency). The major transport protein responsible for the uptake of iron and manganese is divalent metal transporter 1 (DMT1, also known as NRAMP2 and SLC11A2), which exists in two isoforms—DMT1A and DMT1B (Mims and Prchal 2005). The 1A isoform is mostly found in the duodenum, whereas DMT1B is ubiquitously expressed. Both forms of DMT1 are capable of transporting several divalent cations in a pH-dependent manner and in the respective order of preference Cd^2+^ > Fe^2+^ > Mn^2+^, Co^2+^ ≫ Cu^2+^ (Mackenzie et al. 2007; Illing et al. 2012). Of relevance to PD is the finding that DMT1 and iron staining are tightly correlated in the basal ganglia (Huang et al. 2004). Accordingly, alterations in DMT1 levels may underlie the abnormal accumulation of metal ions in the SN and thereby PD pathogenesis. Supporting this, DMT1 expression has been reported to increase with age (Ke et al. 2005) and in the SNpc DA neurons of PD patients compared to age-matched controls, which correlates with a rise in iron content (Salazar et al. 2008). Further, mice intoxicated with the parkinsonian neurotoxin MPTP also register an increase in DMT1 levels in the ventral mesencephalon concomitant with iron accumulation, oxidative stress and DA neuronal loss (Salazar et al. 2008). Conversely, rodents carrying a mutant DMT1 that impairs transport are significantly protected against MPTP-induced neurotoxicity (Salazar et al. 2008). Together, these studies suggest a role for DMT1 in PD pathogenesis.

To examine the potential role of DMT1 in PD, we have generated transgenic mice overexpressing monkey DMT1B under the direction of a mouse prion promoter, which express in several regions of the brain. When these mice are fed with iron-supplemented diet, they exhibit robust accumulation of iron especially in the SN. Interestingly, the treated mice also display markedly enhanced level of Parkin (but not α-synuclein) that might explain their lack of an overt phenotype by virtue of the neuroprotective role of Parkin. As an extension of this study, we have also generated and characterized DMT1-expressing mice against a Parkin null background. Interestingly, these double-mutant mice continue to resist a disease phenotype even when fed with iron- or manganese-supplemented diet, although they exhibit greater vulnerability toward 6-hydroxydopamine (6-OHDA)-induced neurotoxicity. Taken together, our results suggest that multiple hits are required to promote PD.

## Materials and Methods

### Antibodies

The following antibodies were used: anti-β-actin (Sigma), anti-c-myc (Roche), anti-DMT1 (Abnova), anti-ferroportin (Novus Biologicals, Littleton, CO), anti-GAPDH (Abcam), anti-Parkin clone PRK8 (Covance, Princeton, NJ), anti-α-synuclein (BD Biosciences), anti-TH (Pel-Freez Biologicals, Rogers, AR), anti-transferrin receptor (Abcam) and HRP-conjugated anti-mouse and anti-rabbit (Sigma).

### Generation of DMT1 Transgenic Mice

**Fig. 1:**
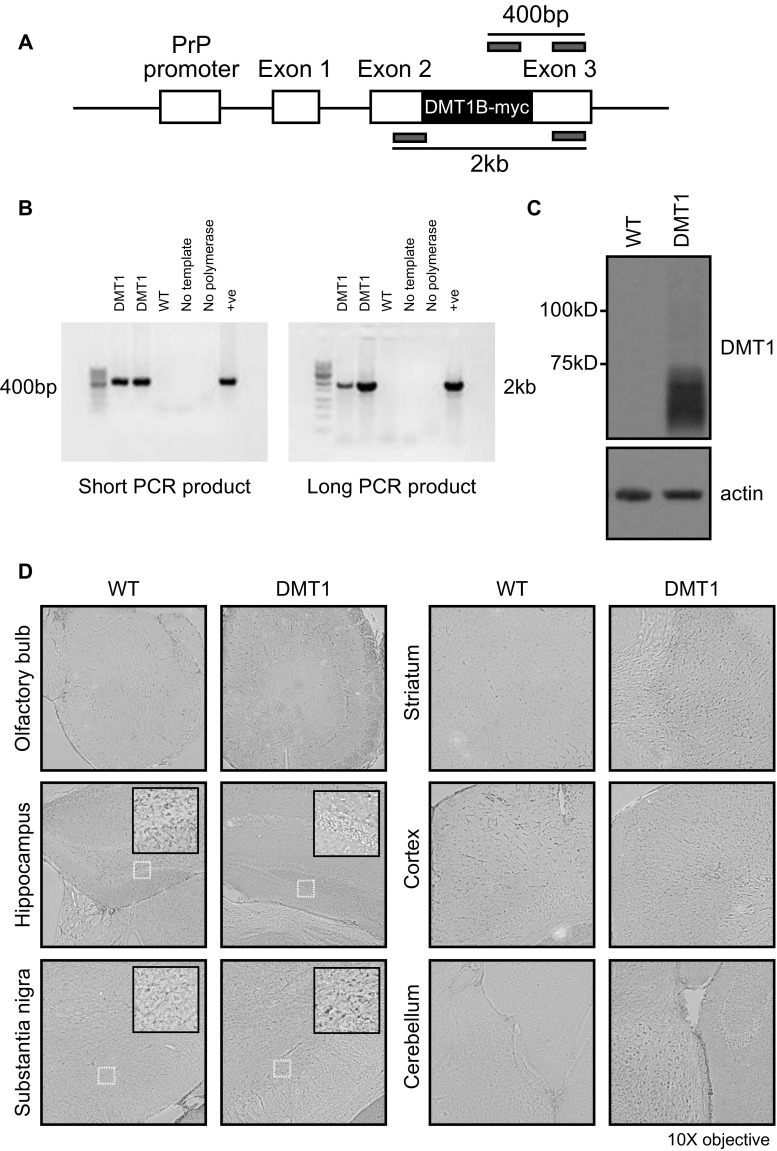
Generation and characterization of transgenic mice overexpressing DMT1. **a** Schematic diagram of the DMT1 construct microinjected into mice. The moPrP promoter was used to drive the expression of DMT1-myc in the brains of mice. *Gray rectangles* show primer pairs used for genotyping, producing either a 2 kb (long) or 400 bp (short) PCR product. **b** Genotyping PCR of DMT1 transgenic mice showing short and long PCR products from three representative mice. No template and no polymerase lanes are negative controls lacking genomic DNA or Taq polymerase enzyme, respectively. **c** Western blot of whole brain lysate from DMT1 transgenic mouse and WT control, detected with anti-DMT1 antibody. β-actin was used as a loading control. **d** Immunohistochemical sections showing DMT1 expression in different regions of WT and DMT1 mouse brains (olfactory bulb, hippocampus, substantia nigra, striatum, cortex and cerebellum) as detected using anti-DMT1B antibody. (*Insets*) Magnified view of highlighted regions inside the hippocampus and substantia nigra

All animal-related work was carried out with the approval and in accordance with the guidelines of the Institutional Animal Care and Use Committee of the National University of Singapore (protocol 008/12) as well as the National Neuroscience Institute (protocol TNI 14-01-001).Previously, we used monkey DMT1 to generate stable cell lines that could take up iron or manganese (Tai et al. 2016). For this work, the cDNA of monkey DMT1 (Accession: AF153279) without the 3′ UTR iron response element (DMT1B/-IRE) was cloned with a myc-tag at the 3′ end. This DMT1B-myc construct was then inserted between exon 2 and 3 of the mouse prion promoter MoPrP.Xho expression vector (Fig. 1a) to drive expression in the brain (Borchelt et al. 1996). The linearized vector was injected into C57BL/C3H mouse eggs as a service provided by the Institute of Molecular and Cell Biology, Singapore. The resulting founder mice were backcrossed to C57BL/6 for six generations. Genotyping involved extracting genomic DNA from mouse tails and carrying out PCR with the following primers: PrP Exon 2 For (CCATTTCAACCGAGCTGAAGCATTCTGCCT) and PrP Exon 3 Rev (GTGGATAACCCCTCCCCCAGCCTAGACC), which gave a 2 kb product, or DMT1B For (CCCTTTGCCCTCATACCCATCCTCAC) with PrP Exon 3 Rev, which gave a 400 bp product. The PCR products were separated on a 1% agarose gel for visualization. The transgene was maintained in the heterozygous form by crossing heterozygous DMT1 mice with WT controls. The DMT1/Parkin KO double-mutant mice were generated by crossing Parkin null mice (Von Coelln et al. 2004) with DMT1 mice. Mice were maintained on a 12-h light/dark cycle with food and water available ad libitum.

### Iron- and Manganese-Supplemented Feed

DMT1 transgenic animals and WT littermates were aged to 9 months on a normal diet (AIN-93M with 35 mg/kg Fe and 10 mg/kg Mn (Reeves et al. 1993)) and then given either normal feed or feed supplemented with 1000 mg/kg of iron (TD110540, Harlan Laboratories) for another 9 months. DMT1/Parkin knockout double-mutant mice were given either normal diet, feed supplemented with 1000 mg/kg of iron or 1000 mg/kg of manganese (TD110541, Harlan Laboratories).

### Western Blotting

Mouse brains were harvested and lysed in PBS buffer containing 1% SDS (w/v) with added protease inhibitor (Roche). Lysates were homogenized using a handheld Teflon glass homogenizer for 15 strokes on ice and then sonicated for 15 s. Centrifugation was then carried out at 13,500 rpm for 15 min and the supernatant collected. Protein content was determined using the Bradford assay (Pierce). Approximately 55 to 100 µg of total protein was resolved on 10–15% SDS-PAGE gel before transferring onto PVDF membrane (Millipore). Detection was carried out using ECL reagent (Pierce).

### Histology

Mice were anaesthetized and transcardially perfused with saline (0.9% NaCl, w/v) followed by 4% PFA (w/v). The brains were removed and post-fixed for another 24 h before placing into cryoprotectant containing 15% sucrose (w/v) in PBS for 24 h and then 30% sucrose for another 24 h. Brains were sectioned in the coronal plane at 20 µm thickness and mounted on histology slides (Marienfeld, Germany). Primary and secondary antibodies were diluted in blocking buffer containing 0.5% BSA (w/v), 0.5% FBS (w/v) and 0.1% Tween 20 (v/v) in PBS. Primary antibody incubations were done overnight at 4 °C while secondary antibodies were incubated for 30 min at room temperature. Sections were washed three times after primary and secondary antibodies with TNT wash buffer consisting of 0.1M Tris-HCl (pH 7.5), 0.15M NaCl and 0.05% Tween 20 (v/v). For immunohistochemistry, antibody signals were amplified with VectorElite ABC Kit (Vector Laboratories) and developed using DAB (Vector Laboratories) according to the manufacturer’s protocol. The sections were then dehydrated under alcohol gradient (75, 90 and 100%) and subsequently two changes of xylene for 3 min each. The sections were then mounted using DPX mounting medium and viewed using a BX61 microscope (Olympus).

### Perls Prussian Blue Stain

Fixed brain sections were immersed in Perls solution consisting of 2% HCl (v/v) and 2% potassium ferrocyanide (w/v) in a 1:1 ratio for 30 min at room temperature. Sections were washed and counterstained with Nuclear Fast Red solution (Sigma) for 5 min. The sections were dehydrated under alcohol gradient (75, 90 and 100%) followed by two changes of xylene. Finally, the sections were mounted using DPX and viewed with a BX61 microscope (Olympus).

### T2-Weighted Magnetic Resonance Imaging (MRI)

Mice were anesthetized with ketamine/xylazine (10 mg/kg) and transcardially perfused with saline (0.9% NaCl, w/v) and thereafter with 4% PFA (w/v). The brains were then harvested and embedded in 1% agarose. T2-weighted MRI was acquired on a 9.4 Tesla scanner (Agilent Technologies, Santa Clara, CA) using a volume transmit/receive coil and the following parameters: multislice fast spin echo; 100-µm isotropic resolution; 256 × 192 × 192 matrix size; 25.6 × 19.2 × 19.2 mm^3^ field-of-view; 2000 ms TR; 8 ETL; 39.15 ms effective TE; and 1 average.

### Immunohistochemistry and Stereological Assessment of TH-Positive Neurons

Mice were given a lethal overdose of anesthesia and were perfused through the heart with cold saline, followed by 4% PFA (w/v). Brains were collected, post-fixed overnight and transferred to a 15% and then 30% sucrose solution overnight. Every fourth section (40 μm) through the perfusion-fixed ventral midbrain was collected on CM3050S cryostat (Leica Biosystems) and placed in free-floating PBS. Endogenous peroxidase activity of each section was quenched with 0.3% H_2_O_2_ (Sigma–Aldrich) for 30 min, followed by washing with TNT buffer. Brain sections were then blocked with TNT blocking buffer containing 5% normal goat serum (Jackson ImmunoResearch Laboratories) for another 30 min. Subsequently, sections were incubated in rabbit polyclonal anti-TH antibody (Pel-Freez Biologicals, Rogers, AR) in 5% NGS TNT blocking buffer overnight at 4 °C and washed with TNT buffer the following day. Brain sections were then incubated in secondary biotinylated antibody (Vector Laboratories) for 1 h prior to incubation with *ABC* reagent kit (Vector Laboratories) for 30 min at room temperature. TH+ cells were made visible via DAB staining (Vector Laboratories) before the sections were mounted on glass slides for visualization. To analyze the TH+ neurons in the left and right pars compacta regions of each brain section, unbiased stereological methodology was employed using a computer-assisted system consisting of Axio Imager 2 microscope (Carl Zeiss), equipped with a motorized ASI MS-2000 stage (Applied Scientific Instrumentation, Eugene, OR), a IEEE 1394 camera (Imi Tech, Encinitas, CA) and interfaced with Stereologer software (Stereology Resource Centre, St. Petersburg, FL).

### Measurement of Iron in Urine and Feces

Iron concentrations in the urine and feces were measured using the QuantiChrom Iron Assay Kit (BioAssay Systems, Hayward, CA) according to the manufacturer’s protocol. Air-dried feces were suspended in 1 ml of water for an hour, and the resulting suspension assayed in a 96-well plate. Urine samples were analyzed directly without further dilution. Final reading was measured with a plate reader at 590 nm (Tecan, Switzerland).

### Parkin Real-time PCR and Stable Cell Lines

PCR for Parkin and GAPDH mRNA levels, as well as generation of vector and Parkin overexpression SH-SY5Y stable cell lines have previously been described (Wang et al. 2005).

### Generation of 6-OHDA Lesion Mouse PD Model and Apomorphine-Induced Rotation Assessment

Mice of 4–6 months of age were anesthetized with ketamine/xylazine and mounted on a stereotaxic frame (Narishige, Japan). A small hole was drilled in the skull with coordinates AP +0.5 mm to Bregma, ML 2.0 mm left to the midline. 3 ul of 2.5 ug/ul 6-hydroxydopamine (6-OHDA, Sigma–Aldrich) was delivered to the striatum with a Hamilton syringe. Once injection was complete, the needle was left in place for 5 min to allow the pressure of the injected volume to dissipate. The needle was then slowly retracted and the wound closed by suturing. 6 weeks after 6-OHDA injection, the apomorphine-induced rotation assay was carried out. Apomorphine (Sigma–Aldrich) was dissolved in saline with 0.1% ascorbic acid and administered by IP injection into the mouse (1.5 mg/kg). 5 min after apomorphine administration, the mouse was transferred to a circular container and the number of rotations made in 3 min was counted.

### Rotarod Assay

Motor performance assays were carried out using a rotarod (Ugo Basile, Italy). Briefly, the mice were placed on the rotarod with a rotating speed of 4 rpm. Once the mice were ready on the beam, the speed was accelerated to 40 rpm within 4 min and the time spent on the rotarod was recorded. Each mouse was given three trials with 10–15-min rest after every trial.

### Statistical Analysis

All data are expressed as mean ± S.E.M. Statistical analysis was performed using Student’s two-tailed *t* test, one-way or two-way ANOVA and statistical significance was considered when *p* < 0.05.

## Results

### DMT-1 Expressing Mice Exhibit Iron Accumulation in the Brain

To generate transgenic mice that overexpress DMT1, we cloned the cDNA of monkey DMT1 into the MoPrP.Xho expression vector (Fig. 1a), which drives high expression of the transgene in most CNS neurons via the murine prion promoter. Transgenic founders were backcrossed for six generations, and the DMT1 transgene was subsequently maintained in the heterozygous state. Analysis of transgene genotype via PCR (Fig. 1b) and protein immunoblotting (Fig. 1c) show the presence and robust expression of DMT1 transgene in the brains of these mice. Consistent with this, strong DMT1 immunoreactivity was observed in all the six brain regions surveyed, i.e., olfactory bulb, hippocampus, substantia nigra, striatum, cortex and cerebellum, relative to their control counterparts (Fig. 1d).

**Fig. 2:**
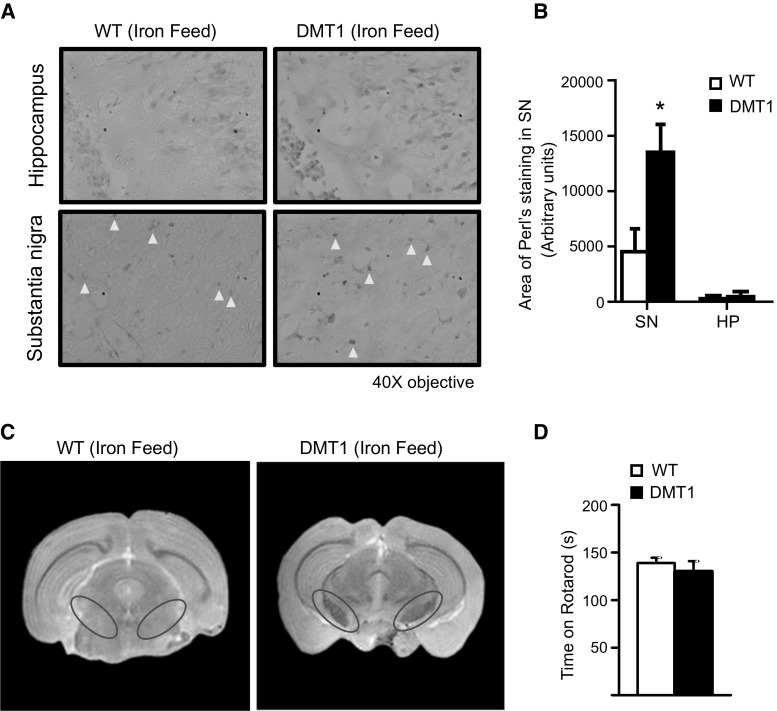
DMT1-expressing mice fed with iron-supplemented diet exhibit robust iron accumulation. **a** WT or DMT1 transgenic mice were aged for 9 months on normal diet and then put on a 1000 mg/kg iron-supplemented diet for another 9 months. At 18 months of age, mouse brains were harvested and Perl’s staining of substantia nigra and hippocampal sections was carried out. Regions of blue Perl’s staining are indicated by *yellow arrowheads*. **b** The *associated bar graph* shows the area of Perl’s staining in arbitrary units in the substantia nigra and hippocampal sections of WT and DMT1 mice, represented as mean ± S.E.M. *p < 0.05, *n* = 4, Student’s *t*-test. **b** Coronal brain section of T2-weighted MRI from WT control or DMT1 transgenic mice aged for 9 months and then given a 1000 mg/kg iron-supplemented diet for another 9 months. Substantia nigra of each brain section is *circled* in *red*. **c** Rotarod performance measuring time spent on the beam in 240-s trials (*n* = 5), with data represented as mean ± S.E.M

Next, we treated 9-month-old DMT1 transgenic mice and their wild-type (WT) littermates with iron-enriched diet (1000 mg/kg) for 9 months to investigate how prolonged iron supplementation together with the aging process influences brain iron accumulation in these mice. We have chosen this treatment paradigm as DMT1-expressing mice fed with normal diet do not exhibit remarkable iron accumulation (data not shown). We first detected for the presence of ferric ions (Fe^3+^) via Perls Prussian blue staining, specifically in the SN where iron is known to accumulate. As anticipated, the level of Perls staining is significantly increased in the SN of DMT1 transgenic mice compared to WT controls (Fig. 2a, b). Comparatively, the difference in Perls staining in the hippocampus between the two groups of mice is not remarkable (Fig. 2a, b). We also carried out a T2-weighted magnetic resonance imaging (MRI) to detect for iron in the brain. Iron has paramagnetic properties, and its abnormal deposition in the brain alters the homogeneity of the magnetic field, enhancing tissue T2 relaxation. Thus, changes in T2 relaxation (T2 shortening) as measured by MRI are associated with iron brain accumulation. This is detected as a decrease in signal intensity of T2-weighted images, called T2 hypo-intensity (Garrick and Garrick 2009). T2-weighted MRI scan revealed a significant reduction in signal intensity in the SN of DMT1 transgenic mice relative to their non-transgenic counterparts (Fig. 2c), which supports the enhancement of iron accumulation in this region. We also analyzed iron excretion in urine and feces of these mice and found that DMT1 mice have reduced iron excretion compared to WT mice for both control and iron-supplemented feed (Fig. S1A, B). While iron excreted in urine by WT mice increased with iron-fed supplementation, the amount excreted by DMT1 mice did not increase as much, suggesting that the additional iron from the supplemented feed is being retained in DMT1 mice. Importantly, both DMT1 and WT mice are of similar weights (Fig. S1C), suggesting that the differences in iron excretion are unlikely to be due to greater feed consumption by WT mice. To rule out the possibility that DMT1 overexpression might affect the level of other iron transport proteins, we measured the expression of transferrin receptor (TFRC) and ferroportin (FPN) via immunofluorescence and found no significant difference in their levels between DMT1 and WT mice fed with iron-supplemented diet (Fig. S2).

### Parkin is Upregulated in the Brain of DMT1-Expressing Mice

**Fig. 3:**
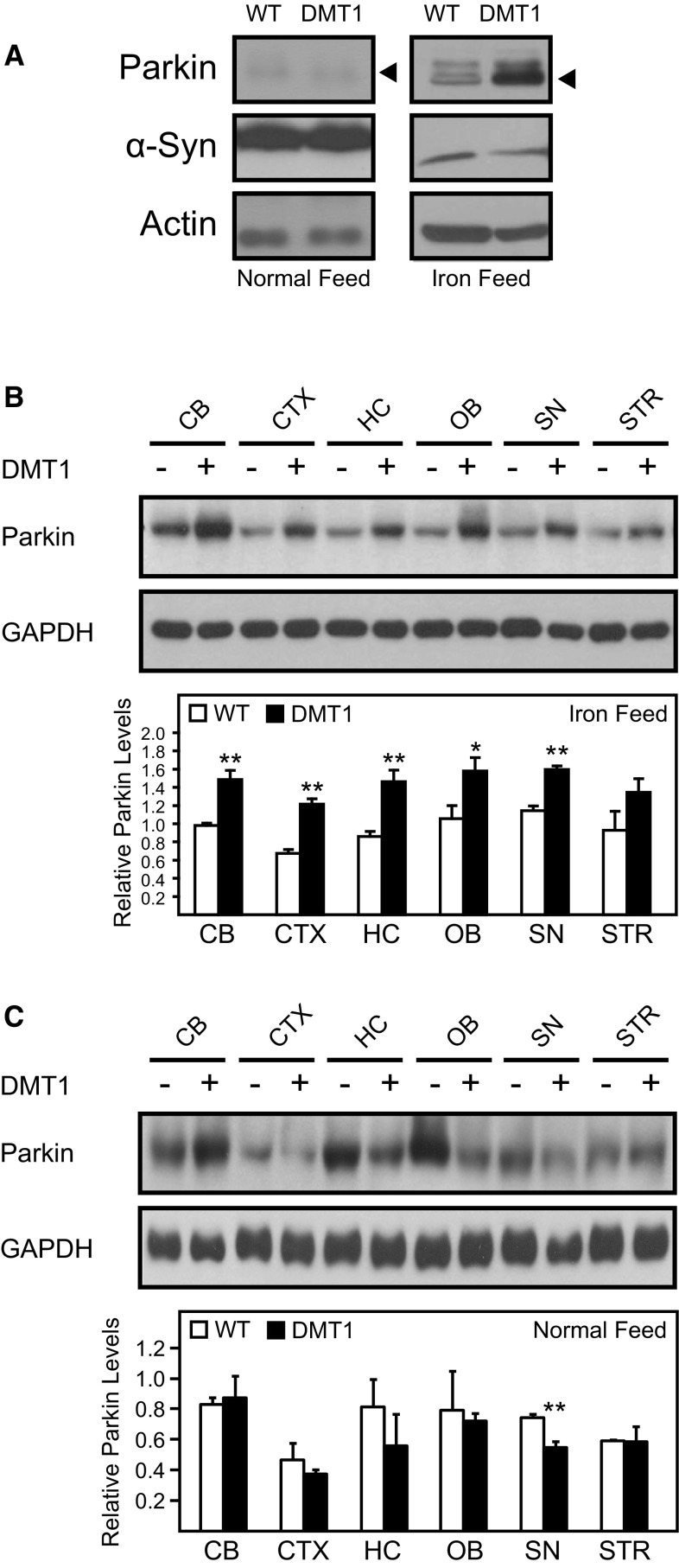
Upregulation of Parkin in control and DMT1-expressing mice fed with iron-supplemented diet. **a** Representative western blot showing the expression of Parkin in the brains of WT and DMT1 mice following 12 months of feeding with either normal or iron-enriched feed. **b**, **c** Representative western blots showing Parkin expression level in the cerebellum (CB), cortex (CTX), hippocampus (HC), olfactory bulb (OB), substantia nigra (SN) and striatum (STR) of WT and DMT1 mice following 12 months of feeding with either iron-enriched (**b**) or normal diet (**c**). *Corresponding bar graphs* show Parkin quantification, with data represented as mean ± S.E.M. * *p* < 0.05; ** *p* < 0.01, *n* = 3, Student’s *t*-test

Although the level of iron is dramatically increased in the SN of DMT1-expressing mice fed with iron-supplemented diet, it does not appear to compromise the function of nigral dopaminergic neurons as the motor function of these mice as measured by the rotarod assay remains largely unaffected (Fig. 2d). Notably, we have previously reported that treatment of cells with various stressors, including iron, promotes the expression of the neuroprotectant Parkin (Wang et al. 2005), an observation that we have reproduced in this study with SH-SY5Y cells (Fig. S3A, B). Consistent with the neuroprotective role of Parkin, we further found that SH-SY5Y cells stably expressing Parkin are protected against iron-mediated toxicity compared to control cells (Fig. S3C, D). Given these, we wondered whether the same phenomenon is occurring in vivo in the case of DMT1-expressing mice. Indeed, immunoblotting of whole brain lysates revealed a dramatic increase in Parkin expression in DMT1 transgenic mice compared to WT controls that are fed with iron-supplemented diet (Fig. 3a). In contrast, the expression of another PD-linked gene, i.e., α-synuclein, remains unchanged between the two groups of mice fed on the same diet (Fig. 3a). The increase in Parkin expression in iron-supplemented DMT1 mice versus control is observed virtually throughout the brain regions examined, including the cerebellum, cortex, hippocampus, olfactory bulb and substantia nigra (Fig. 3b). Comparatively, Parkin expression remains not remarkably affected in the two groups of mice fed with normal diet, except in the SN where the level of Parkin is apparently reduced in DMT1 transgenic mice (Fig. 3c). Why a selective reduction in Parkin expression is observed in the SN of these transgenic mice fed with normal diet is unclear to us at the moment. Nonetheless, our results collectively suggest that Parkin expression is increased in iron-supplemented DMT1-expressing mice presumably as a neuroprotective response to iron accumulation.

### Generation and Characterization of DMT1/Parkin Knockout Double-Mutant Mice

**Fig. 4:**
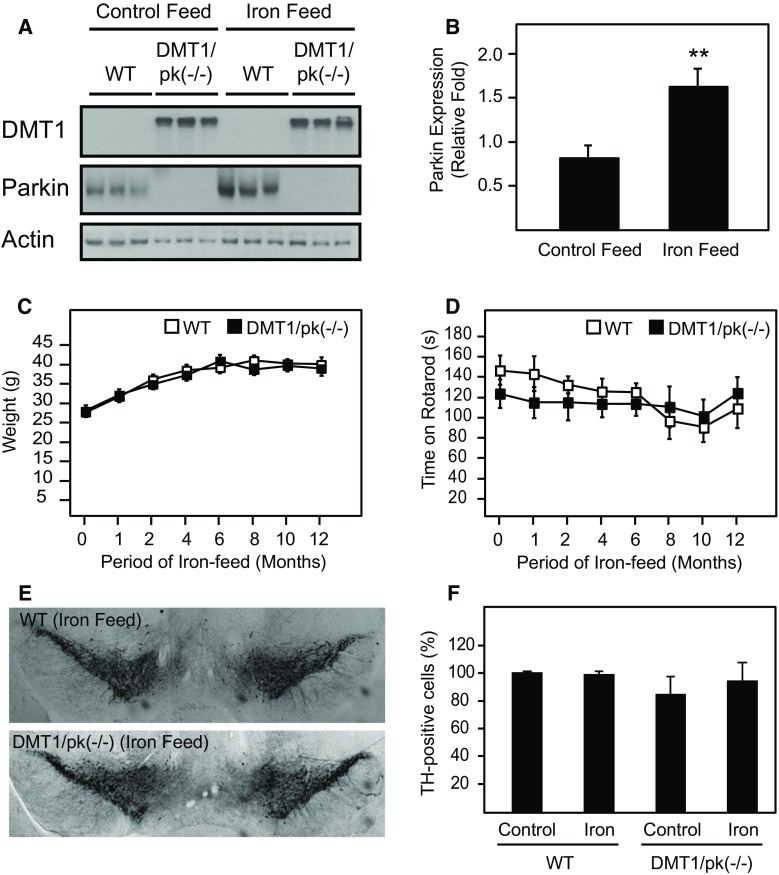
Histological and behavioral characterization of DMT1/Parkin KO double-mutant mice given either normal or iron-enriched diet. **a** Representative western blots of DMT1 and Parkin expression in the brains of WT and double-transgenic mice following 12 months of normal or iron-enriched feeding. **b** Quantification of Parkin expression in the brains of WT mice fed with either normal or iron-enriched feed. Data represented as mean ± S.E.M. ** *p* < 0.01, *n* = 3, Student’s *t*-test. **c** Weights of WT (*n* = 11) and double-transgenic mice (*n* = 10) throughout the 12-month period on iron-enriched diet. **d** Corresponding rotarod latency of WT (*n* = 11) and double-transgenic mice (*n* = 10) throughout the 12-month period. **e** DAB staining of TH-positive cells in the substantia nigra of WT and double-transgenic mice fed with iron-enriched diet. **f** Quantification of TH-positive cells in the substantia nigra of WT and double-transgenic mice (*n* = 3) following chronic feeding with either normal or iron-enriched food. Data represented as mean ± S.E.M

**Fig. 5:**
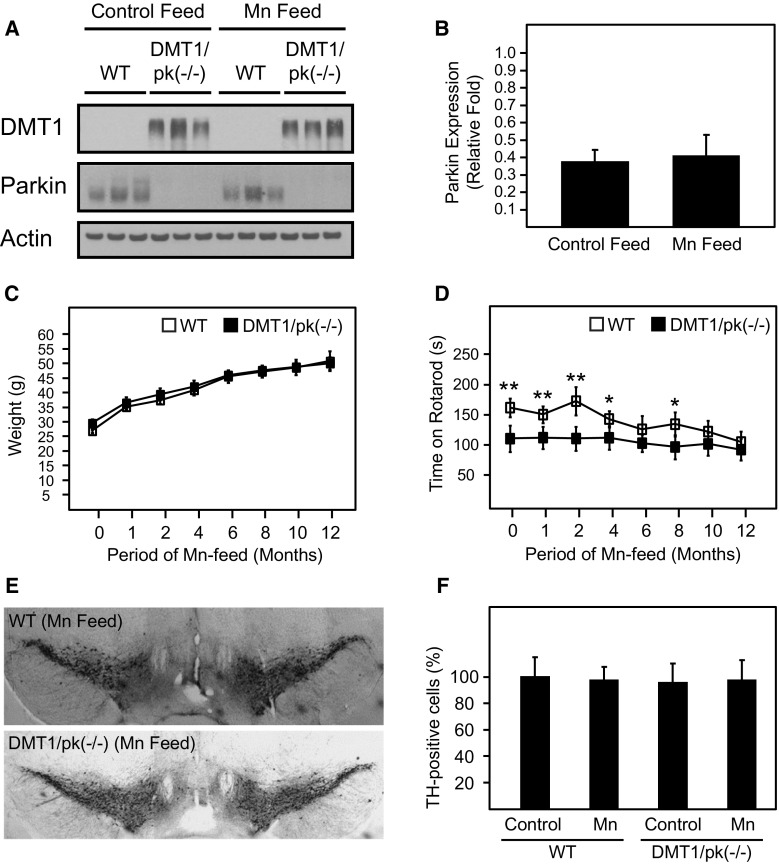
Histological and behavioral characterization of DMT1/Parkin KO double-mutant mice given either normal or manganese-enriched diet. **a** Representative western blots of DMT1 and Parkin expression in the brains of WT and double-transgenic mice following 12 months of normal or manganese-enriched feeding. **b** Quantitation of Parkin expression in the brains of WT mice (*n* = 3) fed with either normal or manganese-enriched feed. Data represented as mean ± S.E.M. **c** Weights of WT (*n* = 12) and double-transgenic mice (*n* = 13) throughout the 12-month period on manganese-enriched diet. **d** Corresponding rotarod latency of WT (*n* = 12) and double-transgenic mice (*n* = 13) throughout the 12-month period. **e** DAB staining of TH-positive cells in the substantia nigra of WT and double-transgenic mice fed with manganese-enriched diet. **f** Quantitation of TH-positive cells in the substantia nigra of WT and double-transgenic mice following chronic feeding with either normal or manganese-enriched food. Data represented as mean ± S.E.M, * *p* < 0.05; ** *p* < 0.01, *n* = 3, Student’s *t*-test

In view of our findings above, we speculated that the ablation of Parkin function in DMT1-expressing mice would likely promote a PD-related phenotype. To address this, we crossed DMT1 transgenic mice with Parkin-deficient mice to generate DMT-expressing mice against a Parkin null background (i.e., DMT1/Parkin KO). Again, we fed these mice with normal or iron-supplemented diet but for a period of 12 months. Unlike control WT mice that exhibit a clear increase in brain Parkin expression with iron-supplemented diet, the expression of Parkin is undetectable in double-mutant mice, as expected (Fig. 4a, b). However, we failed to observe significant differences between DMT1/Parkin KO mice and their control counterparts in terms of growth, rotarod performance and the number of tyrosine hydroxylase (TH)-positive DA cells in the SN (Fig. 4c). As an extension to this, we also examined the effects of a manganese-supplemented diet on the double-mutant mice given that manganese ions can also be similarly transported by DMT1 and that chronic manganese exposure is associated with motoric dysfunction (Marger et al. 2014). Interestingly, we did not observe an increase in Parkin expression in the brain of WT mice fed with manganese-supplemented diet compared to those fed with control diet (Fig. 5a, b), which contradicts a previous study by Higashi and colleague performed in cultured cells (Higashi et al. 2004). Again, no remarkable differences were recorded between DMT1/Parkin KO mice and their control counterparts fed with manganese-supplemented diet in terms of growth and the number of TH-positive cells in the SN, although the double-mutant mice fared significant badly in their rotarod performance in the initial period especially the first four months of manganese-enriched diet (Fig. 5c–f).

**Fig. 6:**
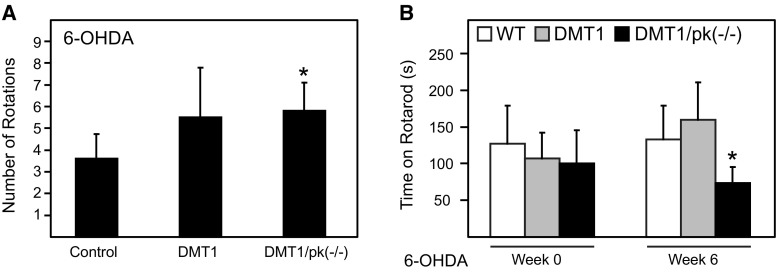
Behavioral characterization of 6-OHDA-lesioned WT, DMT1 and DMT1/Parkin KO double-mutant mice. **a** Bar graph showing the number of rotations by 6-OHDA-lesioned mice following apomorphine challenge. A minimum of three mice aged 4–6 months were used for each group. **b** Rotarod performance measuring time spent on the beam in 240-s trials for 6-OHDA mice at week 0 (*before lesion*) and week 6 (*after lesion*). Data represented as mean ± S.E.M. * *p* < 0.05, *n* = 3, Student’s *t*-test

Finally, we asked the question whether additional stress is needed to promote a pathological phenotype in the double-mutant mice. To address this, unilateral 6-OHDA lesion was carried out in WT, DMT1 and DMT1/Parkin KO animals. When subsequently challenged with apomorphine, the double-mutant mice exhibit significantly more rotational deficits compared to the WT controls (Fig. 6a). Moreover, 6 weeks after challenge with 6-OHDA, the double-mutant mice also exhibit significantly poorer rotarod performance compared to WT (Fig. 6b). Taken together, our results suggest that a combination of stressors is required before overt neurotoxic effects can be seen, which could explain why genetic mouse models of PD tend to lack robust signs of Parkinsonism.

## Discussion

The observation that DMT1 upregulation correlates with iron accumulation in the SN of PD patients (Salazar et al. 2008) has prompted us to investigate the role of the DMT1 transporter in brain metal accumulation and neurodegeneration. For this purpose, we have generated and characterized transgenic mice overexpressing DMT1 in the brain via the murine prion promoter in this study. We found that DMT1-expressing mice express the transgene robustly in various regions of the brain but exhibit rather selective accumulation of iron in the SN when treated with iron-supplemented diet. However, these mice do not display obvious motoric deficits even with age, suggesting that iron accumulation in the SN alone is insufficient to promote DA neurodegeneration. In part, this may be due to the upregulation of Parkin, a potent neuroprotectant, in the brain of these mice. Curiously, DMT1/Parkin KO double-mutant mice treated with iron- or manganese-supplemented also fail to exhibit robust Parkinsonism phenotype, but are nonetheless more susceptible to 6-OHDA-induced neurotoxicity. Taken together, our results support the suggestion that multiple hits are required for DA neuronal loss in PD (Sulzer 2007).

Iron accumulation is widely thought to be detrimental for cellular survival. It is well known that the iron-catalyzed Fenton reaction can covert mitochondrial-related H_2_O_2_ to the highly reactive hydroxyl radical (·OH) that can damage intracellular molecules such as DNA, proteins and lipids. Accordingly, brain regions that have a high burden of iron accumulation are likely to be more susceptible to oxidative stress. The situation is further aggravated in DA neurons as iron can promote the oxidation of dopamine and facilitate the formation of dopamine quinone as well as the neurotoxic 6-OHDA (Hare and Double 2016). Iron can also enhance the aggregation of α-synuclein, which is particularly toxic to DA neurons (Levin et al. 2011). Given these, one could therefore readily appreciate the impact of iron accumulation on DA neuronal survival. However, it remains unclear whether iron accumulation represents a cause or consequence of neurodegeneration in PD (Daugherty and Raz 2015). Notably, several studies have revealed the accumulation of Fe^3+^ (rather than the more reactive Fe^2+^) in the SN of PD brains compared to controls (Dusek et al. 2015). Specifically, Mössbauer spectroscopy can distinguish between Fe^2+^, which generates free radicals via the Fenton reaction, and Fe^3+^ which is usually found stored in ferritin. Using this technique, Galazka-Friedman conducted a study in PD individuals and age-matched controls but found no evidence of Fe^2+^ in the SN, suggesting that iron is stored as the more inert Fe^3+^ form in ferritin (Galazka-Friedman et al. 2012). Similarly, our results showed increased Fe^3+^ in the SN of DMT1 mice compared to WT controls, as detected using Perls staining. Finally, a study that involved MPTP-treated monkeys showed a reduction in TH-positive cells within a week of injection, but increase in iron levels was only observed after 4.5 months (He et al. 2003). This again suggests that iron accumulation may not be a direct cause of neuronal death, and could explain why accumulation of iron is generally observed but not always present in cases of neurodegeneration (Dashtipour et al. 2015). This may also explain why DMT1-expressing mice do not develop any overt signs of disease-associated phenotype despite exhibiting robust accumulation of iron, especially in the SN when fed with iron-supplemented diet. The lack of neurotoxicity in the transgenic mice could also be due in part to the upregulation of Parkin, which was observed in DMT1 mice treated with iron-supplemented diet. Parkin is a multifunctional ubiquitin ligase that is widely regarded to be a broad spectrum neuroprotectant capable of protecting the cells against a plethora of toxic insults (Zhang et al. 2015). Interestingly, a recent study by Roth and colleagues demonstrated that Parkin regulates metal transport via promoting proteasomal degradation of DMT1B (Roth et al. 2010). Consistent with this, Parkin overexpression affords considerable protection to cells treated with manganese (Higashi et al. 2004) and iron (this study). In light of these findings, it was intuitive for us to investigate whether the ablation of Parkin expression would promote a PD phenotype in DMT1-expressing mice. As described above, the double-mutant mice also fail to exhibit robust signs of Parkinsonism, even when treated with iron-supplemented diet until they reached 18 months of age. This reinforces the suggestion that iron accumulation may not be the culprit in PD. Supporting this, we have recently shown that low concentrations of iron can mitigate manganese induced cytotoxicity rather than having deleterious effects (Tai et al. 2016). Unlike iron, chronic manganese exposure is well documented to be the cause of motoric disturbances known as manganism (Chen et al. 2015). Curiously, even when treated with manganese-enriched diet, DMT1/Parkin KO double-mutant mice appear largely normal, although they tend to display lower motor performance on a rotarod in the initial months following treatment. Thus, despite lending itself as a “gene (i.e., Parkin KO)—environmental (i.e., DMT1-mediated accumulation of metal ions)” model of PD, the double-mutant mice are relatively resistant to neurodegeneration. This is somewhat reminiscent of the scenario with the triple Parkin/PINK1/DJ-1 knockout mice, which exhibit no evidence of Parkinsonism (Kitada et al. 2009). Notwithstanding this, DMT1/Parkin KO double-mutant mice are more vulnerable to 6-OHDA-induced neurotoxicity. This appears to be brought about by the combined effects of DMT1 overexpression and Parkin gene ablation as mice expressing DMT1 alone do not exhibit significantly different motoric phenotype from WT mice when treated with 6-OHDA. Similarly, Parkin-deficient mice alone are not more sensitive to the toxin (Perez et al. 2005).

Although many genetic mutations are known to cause or modulate the risk of PD, the low penetrance of some of these mutations together with the low disease concordance in relatives led to the suggestion that there must be interactions among multiple factors for PD to manifest (Sulzer 2007). Our results indicate that a combination of factors is required to promote a disease phenotype in mice and thus support the “multiple hit” hypothesis for the pathogenesis of PD. At the same time, our results may also help to explain why mouse models of PD based on single-gene mutation generally lack robust signs of Parkinsonism.

## Electronic supplementary material

Below is the link to the electronic supplementary material.

Supplementary material 1 (PDF 1059 kb)

## References

[CR1] Borchelt DR, Davis J, Fischer M, Lee MK, Slunt HH, Ratovitsky T (1996). A vector for expressing foreign genes in the brains and hearts of transgenic mice. Genetic Analysis.

[CR2] Chai C, Lim KL (2013). Genetic insights into sporadic Parkinson’s disease pathogenesis. Current Genomics.

[CR3] Chen P, Chakraborty S, Peres TV, Bowman AB, Aschner M (2015). Manganese-induced neurotoxicity: From C. elegans to humans. Toxicol Res (Camb).

[CR4] Dashtipour K, Liu M, Kani C, Dalaie P, Obenaus A, Simmons D (2015). Iron accumulation is not homogenous among patients with Parkinson’s disease. Parkinsons Disease.

[CR5] Daugherty AM, Raz N (2015). Appraising the role of iron in brain aging and cognition: Promises and limitations of MRI methods. Neuropsychology Review.

[CR6] Dexter DT, Wells FR, Lees AJ, Agid F, Agid Y, Jenner P (1989). Increased nigral iron content and alterations in other metal ions occurring in brain in Parkinson’s disease. Journal of Neurochemistry.

[CR7] Dusek P, Roos PM, Litwin T, Schneider SA, Flaten TP, Aaseth J (2015). The neurotoxicity of iron, copper and manganese in Parkinson’s and Wilson’s diseases. Journal of Trace Elements in Medicine and Biology.

[CR8] Galazka-Friedman J, Bauminger ER, Szlachta K, Friedman A (2012). The role of iron in neurodegeneration–Mossbauer spectroscopy, electron microscopy, enzyme-linked immunosorbent assay and neuroimaging studies. Journal of Physics: Condensed Matter.

[CR9] Garrick MD, Garrick LM (2009). Cellular iron transport. Biochimica et Biophysica Acta.

[CR10] Hare DJ, Double KL (2016). Iron and dopamine: A toxic couple. Brain.

[CR11] He Y, Thong PS, Lee T, Leong SK, Mao BY, Dong F (2003). Dopaminergic cell death precedes iron elevation in MPTP-injected monkeys. Free Radical Biology and Medicine.

[CR12] Higashi Y, Asanuma M, Miyazaki I, Hattori N, Mizuno Y, Ogawa N (2004). Parkin attenuates manganese-induced dopaminergic cell death. [Research Support, Non-U.S. Gov’t]. Journal of Neurochemistry.

[CR13] Huang E, Ong WY, Connor JR (2004). Distribution of divalent metal transporter-1 in the monkey basal ganglia. [Comparative Study Research Support, Non-U.S. Gov’t]. Neuroscience.

[CR14] Illing AC, Shawki A, Cunningham CL, Mackenzie B (2012). Substrate profile and metal-ion selectivity of human divalent metal-ion transporter-1. Journal of Biological Chemistry.

[CR15] Ke Y, Chang YZ, Duan XL, Du JR, Zhu L, Wang K (2005). Age-dependent and iron-independent expression of two mRNA isoforms of divalent metal transporter 1 in rat brain. Neurobiology of Aging.

[CR16] Kitada T, Tong Y, Gautier CA, Shen J (2009). Absence of nigral degeneration in aged parkin/DJ-1/PINK1 triple knockout mice. Journal of Neurochemistry.

[CR17] Levin J, Hogen T, Hillmer AS, Bader B, Schmidt F, Kamp F (2011). Generation of ferric iron links oxidative stress to alpha-synuclein oligomer formation. J Parkinsons Dis.

[CR18] Mackenzie B, Takanaga H, Hubert N, Rolfs A, Hediger MA (2007). Functional properties of multiple isoforms of human divalent metal-ion transporter 1 (DMT1). Biochemical Journal.

[CR19] Marger L, Schubert CR, Bertrand D (2014). Zinc: An underappreciated modulatory factor of brain function. Biochemical Pharmacology.

[CR20] Mims MP, Prchal JT (2005). Divalent metal transporter 1. Hematology.

[CR21] Perez FA, Curtis WR, Palmiter RD (2005). Parkin-deficient mice are not more sensitive to 6-hydroxydopamine or methamphetamine neurotoxicity. BMC Neuroscience.

[CR22] Reeves PG, Nielsen FH, Fahey GC (1993). AIN-93 purified diets for laboratory rodents: Final report of the American Institute of Nutrition ad hoc writing committee on the reformulation of the AIN-76A rodent diet. Journal of Nutrition.

[CR23] Roth JA, Singleton S, Feng J, Garrick M, Paradkar PN (2010). Parkin regulates metal transport via proteasomal degradation of the 1B isoforms of divalent metal transporter 1. Journal of Neurochemistry.

[CR24] Salazar J, Mena N, Hunot S, Prigent A, Alvarez-Fischer D, Arredondo M (2008). Divalent metal transporter 1 (DMT1) contributes to neurodegeneration in animal models of Parkinson’s disease. Proceedings of the National Academy Science of USA.

[CR25] Sulzer D (2007). Multiple hit hypotheses for dopamine neuron loss in Parkinson’s disease. Trends in Neurosciences.

[CR26] Tai YK, Chew KC, Tan BW, Lim KL, Soong TW (2016). Iron mitigates DMT1-mediated manganese cytotoxicity via the ASK1-JNK signaling axis: Implications of iron supplementation for manganese toxicity. Science Reports.

[CR27] Von Coelln R, Thomas B, Savitt JM, Lim KL, Sasaki M, Hess EJ (2004). Loss of locus coeruleus neurons and reduced startle in parkin null mice. Proceedings of the National Academy Science of USA.

[CR28] Wang C, Ko HS, Thomas B, Tsang F, Chew KC, Tay SP (2005). Stress-induced alterations in parkin solubility promote parkin aggregation and compromise parkin’s protective function. Human Molecular Genetics.

[CR29] Zecca L, Stroppolo A, Gatti A, Tampellini D, Toscani M, Gallorini M (2004). The role of iron and copper molecules in the neuronal vulnerability of locus coeruleus and substantia nigra during aging. [Research Support, Non-U.S. Gov’t Research Support, U.S. Gov’t, Non-P.H.S.]. Proceedings of the National Academy Science of USA.

[CR30] Zhang CW, Hang L, Yao TP, Lim KL (2015). Parkin regulation and neurodegenerative disorders. Frontiers Aging Neuroscience.

