# Supplementary material for: Transgenic Mice Overexpressing the Divalent Metal Transporter 1 Exhibit Iron Accumulation and Enhanced Parkin Expression in the Brain
Source: Neuromolecular Med. 2017 Jul 10;19(2):375–86. doi: 10.1007/s12017-017-8451-0 (PMC5570798; doi:10.1007/s12017-017-8451-0)
Supplement: Supplementary file 1 — Supplementary material 1 (PDF 1059 kb) [file 12017_2017_8451_MOESM1_ESM.pdf]

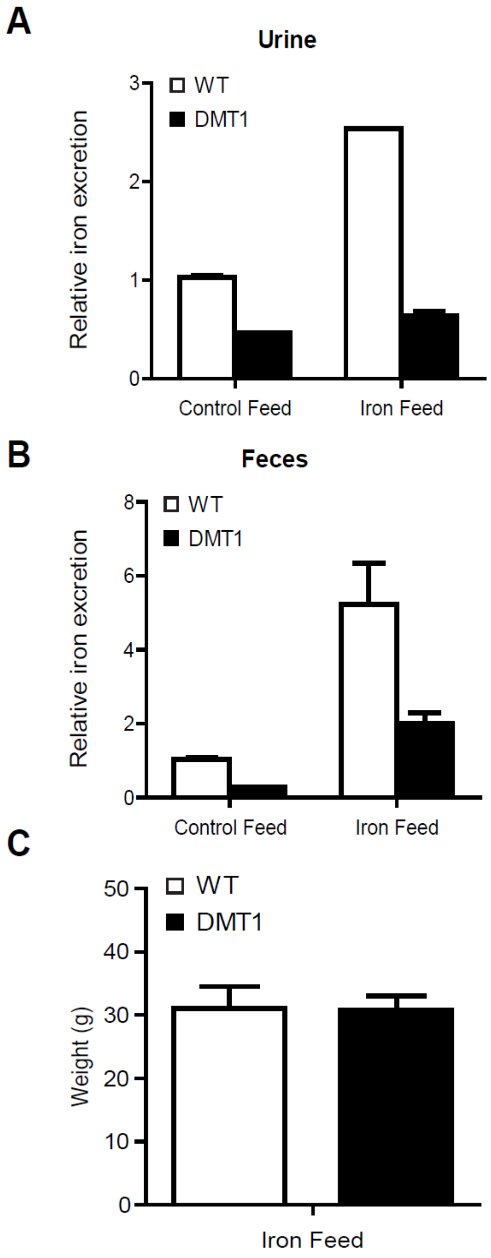

Figure S1. Iron excretion in the urine and feces WT and DMT1 mice. (A) Bar graph showing relative iron excretion in urine from WT (Control=2, Iron=2) and DMT1 mice (Control=2, Iron=4) aged for 9 months and then given an iron-enriched diet for another 9 months. (B) Bar graph showing relative iron excretion in feces from WT (Control=2, Iron=2) and DMT1 mice (Control=2, Iron=4). (C) Weights of 18 month old WT (n=5) and DMT1 (n=7) mice. All data was represented as mean ± S.E.M.

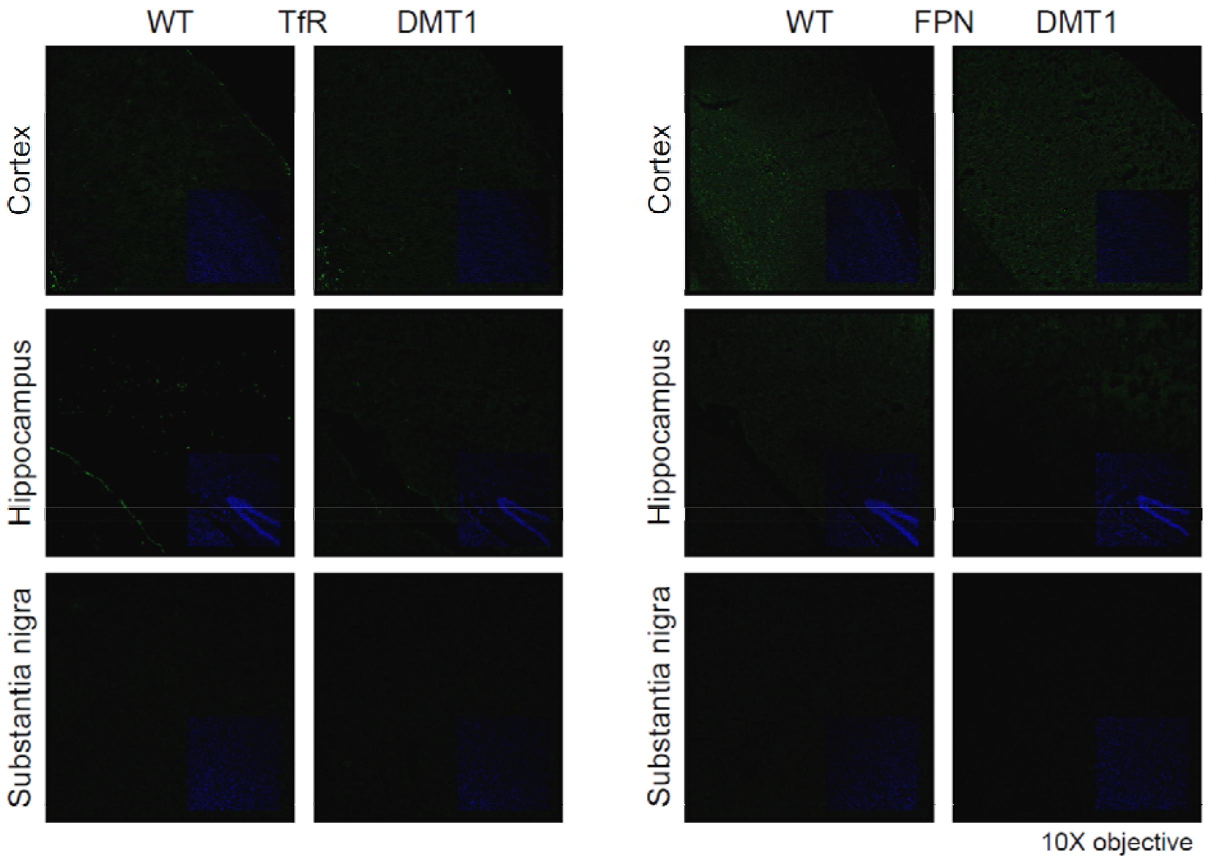

Figure S2. Transferrin receptor (TFRC) and ferroportin (FPN) levels in WT and DMT1 mice given iron supplemented feed. Green immunofluorescence staining of TFRC and FPN in the cortex, hippocampus and substantia nigra of mice aged for 9 months and then given an iron-enriched diet for another 9 months. (Insets) Corresponding blue nuclear staining with Hoechst 33342.

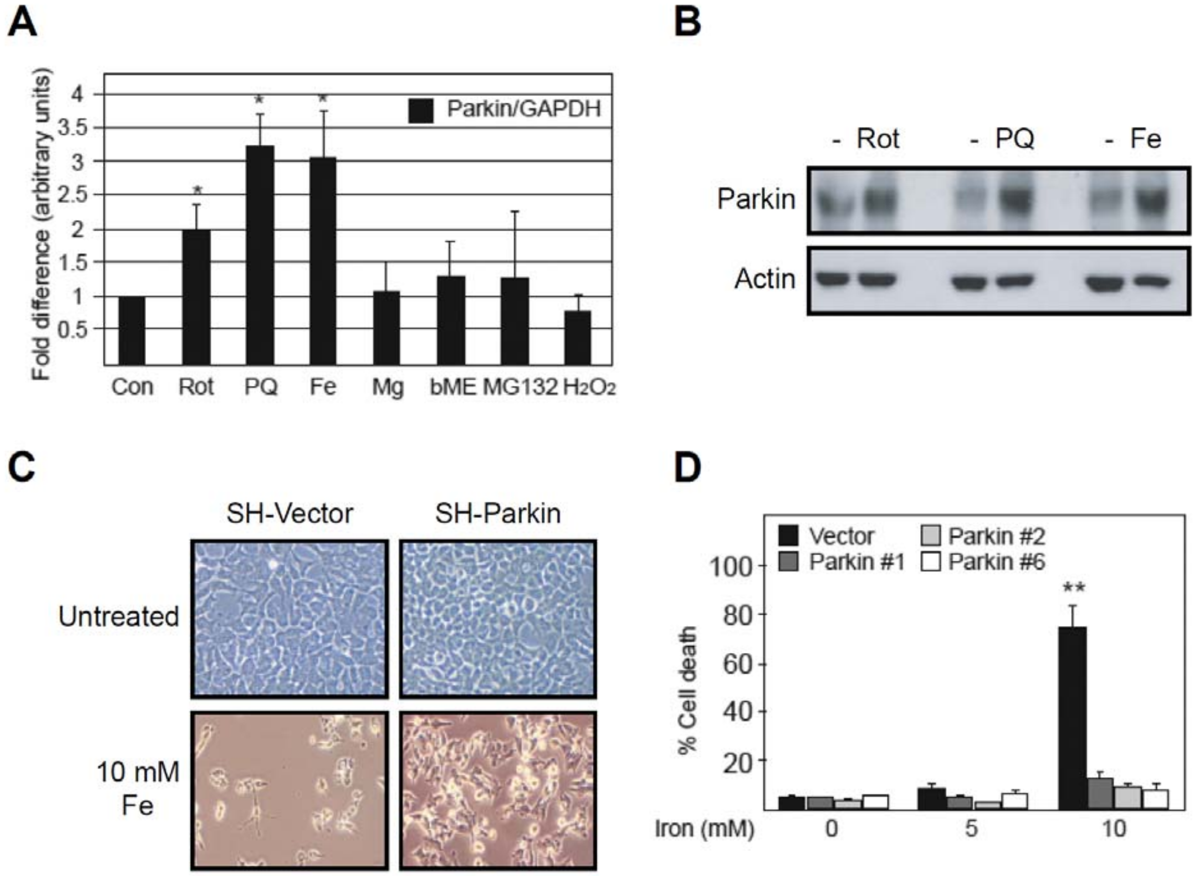

Figure S3. Parkin overexpression protects SH-SY5Y cells from iron-induced toxicity. (A) Bar graph showing the regulation of Parkin mRNA levels in SH-SY5Y cells in response to rotenone (Rot), paraquat (PQ), iron (Fe), manganese (Mn), β-mercaptoethanol (bME), MG132 and H_2O_2 treatments. Data represented as mean ± S.E.M. *$p < 0.05$, n=3, Student's *t*-test. (B) Representative western blot of Parkin protein expression in SH-SY5Y cells in the absence or presence of rotenone, paraquat or iron. (C) Representative phase contrast images of empty vector or Parkin SH-SY5Y stable cell lines with or without 10 mM Fe treatment. (D) Bar graph of percentage of cell death for empty vector or Parkin-transfected SH-SY5Y cells treated with increasing concentrations of iron. Data represented as mean ± S.E.M. **$p < 0.01$, n=3, Student's *t*-test.
